# Unusual Cause of Abdominal Pain

**DOI:** 10.4103/1319-3767.37810

**Published:** 2008-01

**Authors:** S. Khanna, D. Chaudhary

**Affiliations:** Pushpawati Singhania Research Institute for Liver, Renal and Digestive Diseases, New Delhi, India

A 20-year-old male patient presented to the emergency department with severe, constant abdominal pain of 5-day duration without any radiation, vomiting or obstipation. There was no history of alcohol or drug intake, trauma, fever or diarrhea. The patient was non-diabetic and non-hypertensive with no history of sexual contact. On examination, he had tachycardia. There was no tenderness, guarding or rigidity over the abdomen. His blood chemistry was normal.

A plain X-ray of the abdomen was taken [Figure 1]. The patient had not undergone any contrast studies. Subsequently, a colonoscopy was performed [Figure 2]. He was continued on laxatives; 2 days after the colonoscopy, the patient was pain-free.

**Figure 1:**
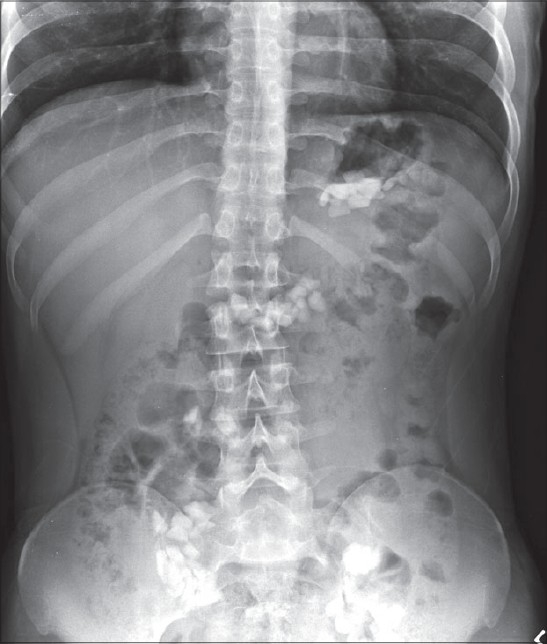
Plain X-ray of the abdomen showing multiple radio-opaque shadows in the colon

**Figure 2:**
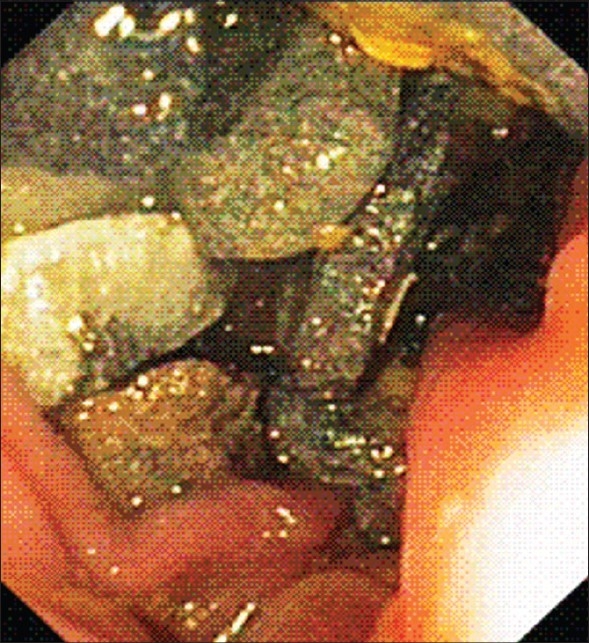
Endoscopic image of the colon

## WHAT IS THE DIAGNOSIS?

## ANSWER

Colon impaction with pebbles - geophagia.

Multiple small stones are seen in the colon. On further history-taking, the patient admitted that he had a habit of ingesting pebbles.

